# Abstract of the Proceedings of the Medical Society of the State of New York, at Its Annual Meeting, in February, 1847

**Published:** 1847-05

**Authors:** 


### *Abstract of the Proceedings of the Medical Society of the State of New York, at its annual Meeting, in February,* 1847.

[CONCLUDED FROM OUR LAST.]

The Committee appointed to propose subjects for the consideration of sectional Committees, reported through their chairman, Dr. Stevens, that the following subjects be embraced within these sections, viz.:—

| Subjects | Committee |
|---|---|
| *First Section.*<br>1. Public Hygyene,<br>2. Statistics of subjects allied to medicine. | Chas. A. Lee, *Chairman.*<br>James McNaughten,<br>J. S. Sprague,<br>C. B. Coventry,<br>P. Van Buren. |
| *Second Section.*<br>1. Medical ethics,<br>2. Medical Education,<br>3. Forensic Medicine,<br>4. Agriculture, | A. Brigham, *Chairman.*<br>N. S. Davis,<br>T. W. Blatchford,<br>Andrew Sill,<br>Alex. Thompson. |
| *Third Section.*<br>1. Lunatic Asylums,<br>2. Public Hospitals,<br>3. Deaf and Dumb Institutions.<br>4. Inst. for the Blind and Idiots,<br>5. Public Dispensaries and Infirmaries, and Public Prisons. | Alex. H. Stevens, *Chairman.*<br>Thomas Spencer,<br>John McCall,<br>T. R. Beck.<br>B. Burwell, |

The gentlemen whose names are placed opposite the sections, were subsequently chosen to take charge of these departments, and present annual reports upon the same to the Society. As the whole State is embraced in the plan, it is confidently believed that a very large amount of useful information, connected with these very important subjects, will thus be annually provided for the profession, as well as the public generally. Increased interest will also thus be given to the meetings of the Society.

A communication was received from the Erie County Medical Society on the subject of admitting students to examination in some of our medical colleges, who have studied with irregular practitioners, which was referred to a committee to report upon the same.

A committee was appointed at the suggestion of Dr. Lee, to confer with the committee of the Legislature appointed to revise and codify the laws of the State, in relation to the medical laws, that they may be reduced to proper order, &c., and Drs. Lee, McNaughten and Wing were chosen on the committee.

Dr. Davis read an interesting practical paper on chronic diseases of the digestive organs, for which the thanks of the Society were given, and the communication referred to the committee of publication.

Dr. Sill, from the committee to procure the use of the Assembly-chamber, for the delivery of the President's address, reported that the chamber would be at the disposal of the Society at four o'clock P. M.

Dr. Blatchford presented to the Society for examination, an instrument for removing elongated uvulæ.

Dr. March presented specimens of malformation, diseased bones, etc., for the inspection of the members. One of these was a unique case of malformation of a fœtus, where the heart was external to the body, lying immediately on the sternum, which was perforated for the transmission of the blood-vessels. Another specimen was the larynx, œsophagus, etc., of an adult who was suffocated by an immense mass of meat lodged in the pharynx, which pressed upon the larynx, and completely closed the rima-glottidis. A third, was a case of double sex, similar to the one reported by Dr. Barry in the last number of our Journal (see page 57). Dr. Stevens and other members offered some interesting remarks relating to different specimens.

On motion, it was resolved, that the editor of the New York Journal of Medicine be permitted to publish an abstract of the proceedings of the Society, at its present session, in his periodical.

On motion of Dr. Stevens, it was resolved, that the members of the Society, be requested to prepare for reading at the next annual meeting a history of the diseases of their respective counties.

At 4 o'clock, P. M., Feb. 3d, the society met, pursuant to adjournment, in the Assembly-chamber, when Dr. McCall, the President, delivered a very able adress on the subject of mental manifestations, as caused by healthy and diseased physical action. Thanks were given for the same, and a copy requested for publication.

The Society then proceeded to ballot for officers for the ensuing year, when the following gentlemen were elected :—T. W. Blatchford, *President*; Alex. Thompson, *Vice President* ; P. Van Buren, *Secretary* ; P. Van Olinda, *Treasurer*.

CENSORS : *Southern District*—A. H. Stevens, E. G. Ludlow, James R. Manley ; *Eastern District*—Wm. Bay, Jon. Eights, Joel A. Wing ; *Western District*—William Taylor, B. Burwell, John Coats ; *Middle District*—J. S. Sprague, A. Willard, Daniel Thomas.

Drs. Gilman, (1st Sen. District,) Dr. Cash, (2d), Brinsmade, (3d), Corliss, (4th), Crowe, (5th), Davis, (6th), Van Buren, (7th), Burwell, (8th), were appointed committee of correspondence.

The following gentlemen were elected permanent members: Drs. W. Parker, E. F. Grant, Alden March, T. B. Hicks, T. Goodsell, N. Winton, G. W. Bradford, A. S. Sprague.

The following gentlemen were elected honorary members : Seth Hastings, of Oneida, and H. H. Childs, of Pittsfield.

Committee of Publication—Drs. T. R. Beck, J. McNaughten, J. A. Wing.

The Committee on Prize Questions and Dissertations, same as last year, viz., McNaughten, Beck, Eights, Wendell, Wing, and Blatchford.

Drs. A. H. Stevens, S. H. French, D. Thomas, R. A. Varick, E. Barnes, E. Delafield, D. R. Pearl, S. M. Crawford, were nominated for permanent members.

Drs. P. B. Brooks, E. Bannister, H. Corliss, and John W. Weed, were recommended to the regents for the honorary degree of Doctor of Medicine.

Such is a very brief and, doubtless, imperfect sketch of the proceedings of the Society, during the three days it continued in session. Besides the interesting discussions to which the resolutions gave rise, a special discussion was held on the last day of the meeting on the subject of nervous diseases, Dr. Stevens in the chair, in which Drs. Wing, Blatchford, Davis, Lee, and others participated. We are happy to be able to publish in our present number, some of the communications made to the Society ; others, for which we have not space in this, will appear in our next.

We congratulate the profession on the evidence presented by the above proceedings of our State Society, that a new medical era is dawning upon us, and that there are signs on every side too obvious to be mistaken, that the reign of quackery and imposture is drawing to a close, and that true science is to prevail in its stead. Let every country society then be resuscitated ; let voluntary associations, in addition, be formed in every county, city, or large town ; let every medical practitioner remember that he has a duty himself to perform; and then the repeal of our medical laws, will prove the greatest boon ever conferred by the State upon our profession.

The plan of dividing the members of the State Society into sections, after the manner of the British Association, is an admirable one, for which we are indebted to the distinguished delegate from the College of Physicians and Surgeons of this city, Dr. A. H. Stevens. If we are not greatly deceived, results are to grow out of this organization of the most important nature, both as respects the public health, the condition of our public institutions, as well as medical education and ethics. The Albany *Argus*, which may be supposed to represent the opinion of intelligent laymen on this point, remarks as follows : "At length, then, the united voice of the medical profession will be heard on subjects appropriate to their proper duties as conservators of the public health ; and we are persuaded that the Legislature will hereafter profit by the information that will be collected in the manner proposed, and that some gross abuses, at which humanity revolts, will be brought to light and corrected."

